# Beef Production Systems with Steers of Dairy and Dairy × Beef Breeds Based on Forage and Semi-Natural Pastures

**DOI:** 10.3390/ani9121064

**Published:** 2019-12-02

**Authors:** Anna Hessle, Margrethe Therkildsen, Katarina Arvidsson-Segerkvist

**Affiliations:** 1 Department of Animal Environment and Health, The Swedish University of Agricultural Sciences, P.O. Box 234, SE-532 23 Skara, Sweden; Katarina.Segerkvist@slu.se; 2 Department of Food Science–Differentiated and Biofunctional Foods, Aarhus University, P.O. Box 50, DK-8830 Tjele, Denmark; Margrethe.Therkildsen@food.au.dk

**Keywords:** animal performance, carcass characteristics, feed intensity, grazing, nature conservation, semi-natural pasture, steer, cross-breeding

## Abstract

**Simple Summary:**

Two-thirds of Swedish beef originates from dairy cows and their offspring, and the vast majority of the calves are of pure dairy breeds. At the same time, European Union, EU, and national agri-environmental payments for grazing biodiverse semi-natural pastures are an important source of income for Swedish beef producers. This study investigated whether beef production from dairy cow offspring based on semi-natural pastures could be improved using beef breed bulls as sires, instead of dairy bulls. Dairy × beef breed steers were compared with pure-bred dairy steers in two different production systems, both based on forage and semi-natural pastures, but varying the birth season, indoor feed intensity, number of grazing periods, and slaughter age. There were no differences between the breeds in feed efficiency or liveweight gain during rearing, but there were differences in carcass weight and composition. Based on these results, we concluded that higher-quality carcasses could be obtained if beef breed sires were used in production systems with semi-natural pastures.

**Abstract:**

This study compared animal performance and carcass characteristics in steers born to a dairy breed dam and a dairy or beef breed sire allocated to one out of two production systems, both including grazing semi-natural pastures during summer. Spring-born groups comprising 16 purebred dairy (D) steers and 16 dairy × Charolais crossbreeds (C) were allocated to moderately high indoor feed intensity with slaughter at 21 months of age (H), while two corresponding autumn-born groups of 16 D and 16 C animals were allocated to low indoor feed intensity with slaughter at 28 months of age (L). The animals were mainly fed grass-clover silage while housed. The H steers grazed semi-natural pastures for one summer, whereas the L steers grazed semi-natural pastures for two summers. From weaning to slaughter, liveweight gain was 0.94 and 0.77 kg day^−1^ for H and L steers, respectively (*p* < 0.0001), with no breed effect on weight gain. However, C carcasses had a higher weight, conformation score, and proportion of high-valued retail cuts than D carcasses (*p* ≤ 0.004). Moreover, conformation differed more between LC and LD than between HC and HD. From this study on forage and pasture-based beef production, we concluded that breed had no effect on the performance of living animals, but that dairy × beef crossbred steers produced heavier and higher-quality carcasses than pure-bred dairy steers.

## 1. Introduction

Semi-natural pastures include diverse landscape types, such as open grasslands, tree pastures, limestone plains, and seashores, all of which have been used continuously for fodder production for centuries [1]. These landscapes are characterized by a specific native flora and fauna and are dependent on human management to be maintained [1]. The production value of these areas is lower than for arable land, and the main reasons for continuing to manage these grasslands are the ecological aspects, as they contain many endangered species within several groups of organisms and also performing ecosystem services [1,2]. They also hold traces of cultural heritage values, recreation possibilities, and amenity values [1]. Unfortunately, all over Europe, semi-natural pastures are being threatened, and the greatest single threat is a cessation of grazing and subsequent abandonment [1,2,3]. These areas cannot deliver any other human food than through grazing livestock and are therefore valuable for global food supply [4].

To promote the management of semi-natural pastures, farmers in many European countries receive economic support to maintain grazing livestock in these areas [5,6,7]. This agri-environmental payment makes extensive beef production on semi-natural pastures more financially competitive than indoor rearing and grazing on cultivated leys. The payment usually comprises a considerable proportion of income in Swedish beef production enterprises, especially those based on grazing pastures categorized as having high biological values, which attract a higher payment (2800 SEK ha^−1^) than pastures with general values (1000 SEK ha^−1^) [7,8,9].

As in the other Nordic countries, a high proportion of Swedish beef production is based on dairy cattle, 60% [10]. Following tradition, a vast majority of Swedish dairy cows (89% in 2017) are crossed with bulls of the same breed, but the proportion of beef breed semen doses used on dairy cows has almost quadrupled in the past five years [11,12]. Calvings are evenly distributed over the year [11]. Swedish male calves from dairy cows are most often raised as intact bulls in intensive indoor systems. Only 22% are castrated and raised as steers, often on semi-natural pastures [10], where steers are more suitable than intact bulls due to the low nutrient availability and possible encounters with the public. A problem with dairy steers is their low carcass conformation score [10]. If carcass quality can be increased using beef breed sires, then rearing male calves from dairy cows as steers in grazing systems using semi-natural pastures can result in increased income in beef production. Extensively reared steers, grazing two summers, bring twice the agri-environmental payment of steers grazing for one summer only. On the other hand, a more intensive one-year-grazing system with a lower slaughter age contributes less to global warming potential through lower lifetime feed consumption and enteric methane emissions [13].

The superiority of dairy × beef crossbreeds compared to pure-bred dairy cattle has been established in previous studies, but mainly in intensive rearing conditions [14,15,16]. Eriksson et al. [17] show that the breed effect of using beef breed sires for dairy cows is greater in intensive than extensive production systems, as the genetic potential of the beef breed can be utilized in the former. The effect is reported to be more pronounced for late-maturing breeds, such as Charolais, than for early-maturing breeds, due to the ability of late-maturing breeds to deposit a higher proportion of muscle instead of fat [18]. Hence, studies are needed on the potential benefits of the expanded use of beef breed crosses under low-intensity rearing conditions, where grazing of biodiverse semi-natural pastures is an important part of the system.

The starting hypothesis in this study was that steers originating from dairy cows and descendent from beef breed sires show better production performance than steers from dairy sires, even in low-intensity systems with the grazing of semi-natural pastures. Therefore, the objective of the study was to compare animal performance and carcass characteristics in steers originating from dairy cows crossed with a dairy or a beef breed sire in two different production systems, with varying birth season, indoor feed intensity, and slaughter age, and both including the grazing of semi-natural pastures.

## 2. Materials and Methods

### 2.1. Experimental Design

The experiment was conducted at the Götala Beef and Lamb Research Centre, Skara, in south-western Sweden (58°42′ N, 13°21′ E; elevation 150 m asl.). The Ethics Committee on Animal Experiments in Gothenburg approved the protocol and execution of the study (case number 187-2014). During indoor periods, the animals were kept and fed in groups of four in pens with fully slatted floors, and during grazing periods, all animals were grazing semi-natural pastures.

**Figure 1:**
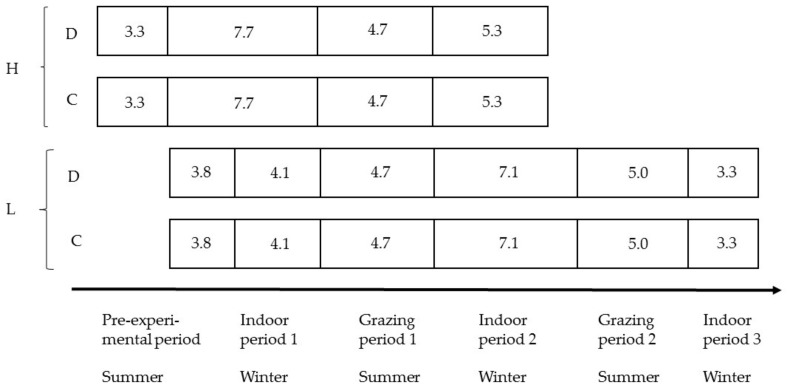
Time schedule in months of the experiment with purebred dairy (D) and cross-bred dairy × beef (C) steers. Spring-born cattle of both breeds were kept on a high feed intensity during indoor periods and slaughtered at 21 months of age (H), while autumn-born cattle of both breeds were kept on a low feed intensity doing indoor periods and slaughtered at 28 months of age (L). In total, there were four treatment combinations of breed and production system (DH, CH, DL, CL), where DH and CH grazed one summer, and DL and CL grazed two summers, all on semi-natural pastures.

The animals were followed from weaning to slaughter in an experiment with a 2 × 2 factorial design, comparing two breeds (dairy, D, and dairy × beef, C) and two production systems (moderately high, H, and low, L, indoor feed intensity; Figure 1). The two production systems differed not only in indoor feed intensity but also in birth season, slaughter age, and the number of summers on the grass. Animals were continuously introduced into the experiment according to birth date, with pairs of calves from the two breeds introduced simultaneously. Hence, the age of individual calves and calves in pens was matched pairwise across breeds (Figure 1). Animals in a pair of pens, i.e., four dairy steers and four crossbreed steers, were slaughtered simultaneously when the average age reached the target age.

### 2.2. Animals

The study included 64 bull calves, all born on the same commercial farm. After the colostrum period, they were housed in small groups of 5–8 calves and fed 5–7 L day^−1^ whole milk up to 2–4 weeks of age, and then they were moved to groups of 15–24 calves and fed milk replacer in a calf-feeder. The calves were offered tender hay and concentrate at ad libitum and were castrated and dehorned at 6–8 weeks of age.

The steers entered the experimental station as weanlings at 3–4 months of age. A total of 32 animals were of the pure dairy breed (D; 12 Swedish Red and 20 Swedish Holstein), and 32 animals were crossbreeds between dairy and beef breeds (C; 12 Swedish Red × Charolais and 20 Swedish Holstein × Charolais). The pure-bred dairy calves descended from 13 different sires (six Swedish Red and seven Swedish Holstein). All crosses were after the same Charolais sire, 3-7238 VB Dennis [19], which was by far the most commonly used bull on Swedish dairy cows during this period, with 33% of all Charolais semen doses from bulls born within a decade before the experiment. Calves in the two breed groups were randomly selected pairwise based on dairy breed (Swedish Red paired with Swedish Red × Charolais and Swedish Holstein paired with Swedish Holstein × Charolais) and birthdate (differing on average by 2.8 ± 2.7 days in the calf pairs). The birth date of the calves ranged from 18 April 2015 to 1 November 2015. During the study, three steers were lost due to death or disease and data from these animals were excluded from further analyses.

### 2.3. Production Systems

Both the D steers and the C steers were split into two sub-groups according to birth date, and the sub-groups were allocated to one of two production systems with varying indoor feed intensity, the number of grazing periods, and slaughter age. Calves born from April to mid-July were allocated to a moderately high feed intensity strategy with slaughter at 21 months of age (H). They had a rather long initial indoor period before they were turned out to pasture at 10–12 months of age. After housing, they were fed an early-cut forage, and they were slaughtered at the end of their second indoor period. Calves born from mid-July to November were allocated to a low feed intensity strategy with slaughter at 28 months of age (L). They were turned out to pasture for a first grazing period at an early age (6–9 months). After housing, they were fed a late-cut forage, allowing only low weight gain. They were then kept on grass for one further grazing period and finally finished indoors on an early-cut forage during their third winter. The set-up with different ranges of birth dates between the H and L groups was due to the need for them to reach both a proper age and weight at first turn-out to pasture and the pre-determined slaughter age within the same indoor period for all animals in a group. The difference in age resulted in the H steers being, on average, older and heavier than L steers during the indoor period 1, grazing period 1, and indoor period 2.

### 2.4. Diets during Indoor Periods

During their first indoor period (Figure 1), all animals were fed ad libitum with a total mixed ration consisting of grass/clover silage, rolled barley, rolled peas, and rapeseed meal, with the protein and energy concentrations adjusted along with increasing live weight according to Swedish nutritional recommendations allowing a liveweight gain of 0.9 kg day^−1^ [20] (Table 1, Table 2 and Table 3). When an average liveweight of 275 kg was reached in a pen, forage was fed ad libitum as the sole feed until slaughter. The forage was grass-clover silage during the remaining part of indoor period 1, and the fully indoor periods 2 and 3 (Table 3), with pasture herbage during the grazing periods in between.

Silage of early-cut grass-clover was taken from the first, second, and third cut, whereas late-cut grass-clover silage was from the first cut only. The herbage contained 90–95% grass (*Lolium perenne, Festuca pratensis, Phleum pratense*, and *Festulolium arundinacea* L.) and 5–10% clover (*Trifolium repens* and *Trifolium pratense*) and it was pre-wilted to about 250 g kg^−1^ dry matter (DM). A preservative containing benzoate, nitrite, methenamine, and propionate was used at 3 L tonne^−1^ herbage (Konsil Ultra, Konsil Scandinavia, Tvååker, Sweden). The herbage was mainly ensiled in bunker silos and harvested at least four months before feeding. At the start and end of the indoor periods, the bunker silage was complemented with round bales of the same cut.

The definition of ad libitum was 105% of voluntary intake. During the indoor periods, the steers were fed once a day, orts were weighed and then disposed of three times a week, and the net average feed consumption per pen was calculated weekly. During the entire experiment, the feed rations were supplemented with vitaminized minerals to fulfill the requirements of the animals [19], and all animals had free access to water and salt.

Silage samples for analysis of chemical composition were taken daily and pooled to one sample per month, while silage samples for analysis of fermentation quality were taken weekly and pooled to one sample per silo or batch of round bales, with three, five, and two samples for indoor period 1, 2, and 3, respectively. Samples of barley, peas, and rapeseed meal were collected weekly and pooled to one sample per feed for analysis of chemical composition.

### 2.5. Grazing Periods

The first grazing period lasted from 2 May to 21 September 2016, and the second grazing period for the L steers lasted from 26 April to 26 September 2017. During grazing period 1, the steers were split across breed and feed intensity into two similar groups, continuously grazing one out of two similar enclosures at a stocking rate of 2.25 animals ha^−1^. The steers in one of the enclosures were infected by the gastrointestinal nematode *Ostertagia ostertagi* [21]. During grazing period 2, the remaining half of the animals, the L steers, were kept in one group, rotating among three enclosures every second to third week, with a total stocking rate of 1.14 animals ha^−1^. The steers were fed a vitaminized mineral supplement ad libitum throughout the grazing periods.

The pasture consisted of 28 ha of permanent semi-natural grassland, with approximately 20% dry, 60% mesic, and 20% wet areas. The pasture was mainly open but included small areas of mixed deciduous trees. The dominant plant species was *Deschampsia cespitosa* (tufted hairgrass), but *Festuca rubra* (red fescue) was also prominently present. In dry areas, *F. ovina* (sheep’s fescue), *D. flexuosa* (wavy hairgrass), *Nardus stricta* (matgrass), and several herb species were abundant. Besides *D. cespitosa* and *F. rubra*, herbs were prevalent in mesic areas, while *D. cespitosa* and Cyperaceae (sedges/rushes) were dominant in wet areas.

**Figure 2:**
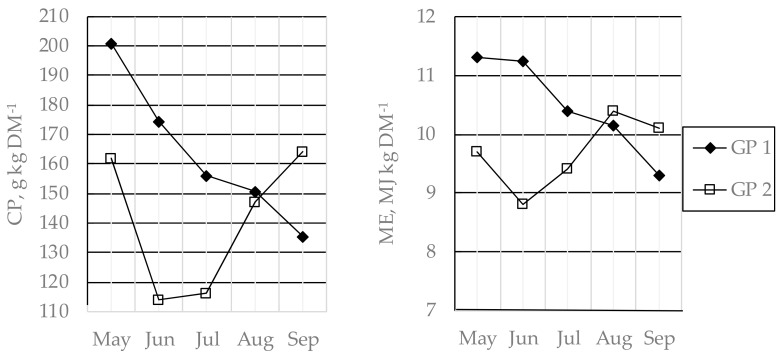

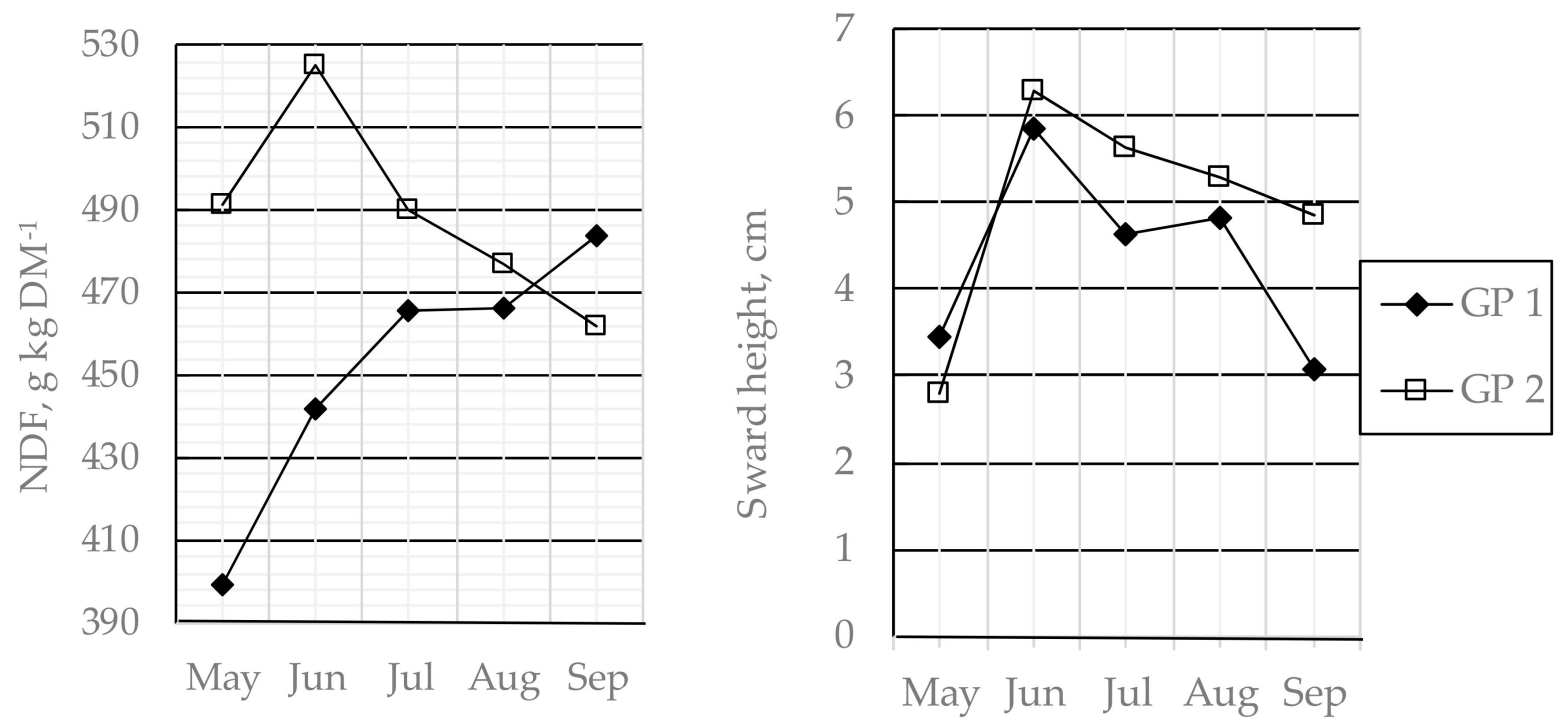
Herbage concentrations of crude protein (CP), metabolizable energy (ME), and neutral detergent fiber (NDF), and sward height in *Deschampsia cespitosa*-dominated semi-natural grassland grazed from May until September by purebred dairy and crossbred dairy × beef steers in grazing period (GP) 1 and 2. Data for GP 1 are based on samples from two enclosures that were continuously grazed, data from GP 2 are average for three rotationally grazed enclosures between entry and exit of grazing animals.

Sward height and chemical composition of the pasture herbage were measured every four weeks from turn-out to housing. In each enclosure, sward height measurement followed a W-shaped route according to Frame [22], with 120–150 recordings performed with a rising plate meter (0.3 m × 0.3 m, weight 430 g). To estimate chemical composition, 25–30 herbage samples were cut with a handheld machine in 3-m diameter circles along the route and pooled to one sample per occasion (Figure 2).

### 2.6. Chemical Analysis

Silage, grain, and pasture samples were analyzed for concentrations of DM, crude protein (CP), neutral detergent fiber (NDF), and in vitro organic matter digestibility. The DM concentrations of silage and pasture were determined at 60 °C and 130 °C, respectively, for 24 h, whereas ash was determined at 550 °C for 5 h. Crude protein was determined according to Dumas [23] and NDF according to Chai and Udén [24]. Metabolizable energy (ME) concentration of silage and pasture herbage was calculated from the in vitro disappearance of rumen organic matter according to Lindgren [25], and ME concentration of grain was calculated according to Axelsson [26]. Concentrations of starch and crude fat were determined in grain [27,28]. In addition, silage samples were analyzed for pH and concentrations of NH_4_-N (Tecator Kjeltec Auto sample system 1035 Analyzer, Tecator Inc., Höganäs, Sweden), organic acids, and ethanol [29].

The chemical composition of the grass-clover silages and concentrates fed during indoor periods is shown in Table 2 and Table 3, while the chemical composition of the pasture herbage is shown in Figure 2.

### 2.7. Carcass Measurements

The steers were slaughtered in a commercial abattoir. The carcasses were divided along the vertebral column and cold carcass weight was estimated as 0.98 × warm carcass weight. Conformation and fat cover were graded according to the European Union Carcass Classification Scheme [30,31].

For conformation, development of the carcass profile, in particular, the round, back, and shoulder was taken into consideration according to the EUROP classification system (E: excellent, U: very good, R: good, O: fair, P: poor). For the fat cover, the amount of subcutaneous fat and fat deposits in the thoracic cavity was taken into account, using a classification range from 1 to 5 (1: low, 2: slight, 3: average, 4: high, 5: very high). Based on the Swedish system [32], each level of the conformation and fatness scales was subdivided into three sub-classes (e.g., O−, O, O+; 3−, 3, 3+) to produce a transformed scale ranging from 1 to 15, with 15 being the best conformation and highest fatness.

After cooling for two days at +2–4 °C, the carcasses were split into the fore- and hind-quarters between the 10th and 11th ribs. Marbling was determined visually on right hindquarters in *musculus (M.) longissimus dorsi* on a 10-point scale with steps of 0.5 from 1 (no marbling) to 5 (slightly abundant), based on the USDA standard [33]. The right hindquarter from each animal was weighed, as were seven high-value retail cuts. These were: strip loin (*M. longissimus dorsi*), fillet (*M. psoas major*), topside (*M. semimembranosus*), outside round (*M. biceps femoris*), eye of round (*M. semitendinosus*), top rump (*M. quadriceps femoris*), and rump steak (*M. gluteus medius*). Trim fat, bones, and two commercially defined cuts from the hindquarter (grade 2 and grade 3 bone-free mincing and stewing meat, assumed to contain 10 and 23% fat, respectively) were also weighed. Trim fat was defined as subcutaneous and intermuscular fat deposits separable with a knife in a standardized cutting procedure. Bones were weighed together with closely bound connective tissue capsules and without extra cleaning of the bones. Dressing proportion (the ratio of cold carcass weight to liveweight), and proportions of retail cuts, trim fat, bones, and the two commercial cuts from the hindquarter were calculated.

### 2.8. Statistical Analysis

Two different statistical models were used, as the dietary intake and feed efficiency data were recorded at the pen level, whereas liveweight gain and carcass characteristics were recorded for the individual animal nested within the pen. To assess the variation in age among animals, relative age was included as a covariate in the models.

Dietary intake and feed efficiency data (Table S1) were analyzed with the Mixed procedure in SAS [34] using the model:

y_ijk_ = µ + α_i_ + β_j_ + αβ_ij_ + c_k_ + cov_ijk_ + e_ijkl_

Liveweight gain and carcass characteristics (Table S2) were analyzed with the Mixed procedure in SAS [34] using the model:

y_ijkl_ = µ + α_i_ + β_j_ + αβ_ij_ + c_k_ + d_l_ + cov_ijkl_ + e_ijklm_

where *µ* is the population mean, *α_i_* is the fixed effect of breed, *β_j_* is the fixed effect of feed intensity, *αβ_ij_* is the interaction between breed and feed intensity, *c_k_* is the fixed effect of parasite infection, *d_l_* is the random effect of pen, *cov_ijk_* and *cov_ijkl_* are the covariate relative age for each individual, and *e_ijkl_* and *e_ijklm_* are the error terms. The variance structure was Profile, and the covariance structure was the Variance component [34]. Only differences between dairy, D, and dairy × beef breed, C, were analyzed, and not the possible breed effects between Swedish Red and Holstein.

Dietary intake data were analyzed separately for each indoor period and summarized over the two or three indoor periods. Liveweight gain data were analyzed for each indoor and grazing period and then averaged over the period from weaning to slaughter. Means were compared pairwise using LSD_0.05_-tests, adjusted by the method of Kenward and Roger [35], and denoted as significant at *p* < 0.05 and as a tendency for significance at 0.05 < *p* < 0.10.

## 3. Results

There was an interaction between breed and production system on the conformation score (*p* = 0.0105), where the CL group had a higher score than CH, whereas DL and DH had the same score. Otherwise, no interaction between breed and production system was found.

### 3.1. Dietary Intake, Liveweight Gain, and Feed Efficiency

Cross-breed C steers were heavier than pure-bred dairy D steers throughout their lifetime, and they had a higher dietary intake expressed as kg DM and kg NDF day^−1^, but not expressed in relation to liveweight (Table 4). Likewise, spring-born moderately high feeding intensity H steers were heavier than autumn-born L steers at the start of indoor period 2 (Table 4), and they had a higher dietary intake than the latter. Feeding the H steers, as planned, with an earlier harvested grass-clover silage than the L steers during the indoor period 2 contributed to an even greater difference (Table 4). However, expressed in relation to liveweight, no effect of the production system on dietary intake was found.

No breed effects on liveweight gain or feed efficiency, expressed as ME MJ kg gain^−1^ were observed. During the first indoor period, the older steers, allocated to the H treatment, tended to have lower feed efficiency than the younger steers allocated to the L treatment. In addition, the H group showed lower weight gain when turned out on pasture (Table 4). However, during the subsequent indoor period 2, the H steers displayed a higher liveweight gain and higher feed efficiency than the L steers (Table 4).

On average, during grazing periods 1 and 2, the pasture stocking rate was 758 and 589 kg liveweight ha^−1^, respectively, and the sward height was 4.3 (SD 2.1) and 4.9 (SD 2.1) cm, respectively. At the end of these grazing periods, average sward height was 3.1 and 4.4 cm, respectively (Figure 2). Infection with pasture-borne parasites affected liveweight gain during the first grazing season (0.43 vs. 0.64 kg day^−1^ for infected and non-infected animals, respectively; *p* = 0.0002). However, there was no other effect of the infection on weight gain during other periods or on the overall liveweight gain from weaning until slaughter.

### 3.2. Carcass Characteristics

Both breed and feed intensity had an impact on the final liveweight at slaughter and carcass weight of the animals. The C steers were heavier than the D steers (682 and 640 kg, respectively), and the L group slaughtered at 28 months of age was heavier than the H group slaughtered at 21 months (695 and 628 kg, respectively) (Table 5). For the C steers, this was also reflected in a higher dressing percentage, whereas there were no differences between the two feed intensity groups H and L. As expected, the C steers had a higher conformation score, and no difference in carcass fatness, but tended to score less in marbling (*p* = 0.054) compared to the D steers (Table 5). The effect of feed intensity had less impact on the conformation score relative to the breed effect (*p* < 0.0844), and there were no effects on either the fatness or marbling score (Table 5). Length of indoor finishing period within feed intensity group (mean 163 ± 23 and 100 ± 34 for the H and L groups, respectively) was positively related to the fatness score (0.4 per month, *p* = 0.0477), but affected no other characteristics.

In the evaluation of carcass composition, the C steers had a heavier hindquarter and higher proportions of the hind-quarter comprising valuable retail cuts and lean meat compared to the purebred D steers (Table 5). On the other hand, the C steers had less bone, whereas there was no difference in the proportion of fat meat cuts or trim fat. Comparisons between the production systems revealed that the hind quarter was heavier in the older L animals, but there were only minor differences in the proportion of valuable retail cuts (*p* = 0.0859). In contrast, the feed intensity had a large impact on the proportion of different meat cuts, with L steers having a larger proportion of lean meat cuts and H steers having a larger proportion of fatty meat cuts (Table 5). This was not reflected in any differences in the amount of trim fat or the bone percentage.

## 4. Discussion

This study examined possible the advantages of dairy cow offspring sired by a late-maturing beef breed instead of a pure dairy breed in production systems involving the use of semi-natural pastures. A significant reason for crossing dairy breed cows with beef breed bulls is to achieve higher capacity for weight gain, but in the present study liveweight gain and feed efficiency were not higher in the C steers than in the purebred D steers. The moderate dietary energy concentration (mainly below 10.5 MJ ME kg^−1^ DM) was most likely too low for the crossbred steers to utilize their higher genetic potential for growth [18,36]. At low dietary energy densities, the maintenance requirement comprises a significant proportion of energy expenditure. As late-maturing beef breeds have a higher maintenance requirement than dairy breeds and early maturing beef breeds, and also lower DM intake capacity, then less energy is available for growth [18,36].

Breed effects on dietary intake in the present study were related to the higher liveweight of the C steers compared to the D steers. At the start (3.3–3.8 months old), the liveweight of the C steers was 25 kg higher than for D steers, and at slaughter this breed difference had increased to 42 kg across the two feed intensities. This result was a surprisingly low increase in difference compared to that in previous studies [14,15,16,37]. However, those studies generally used higher feed intensities, even compared with the production system with a moderately high feed intensity (H) strategy in the present study. A further result of the production system with low feed intensities chosen in this study was a generally low dressing proportion [38], below 50% (Table 5). As these steers were finished on grass-clover silage only, their digesta was most likely more voluminous at slaughter than if a more concentrated feed ration had been fed.

Despite the modest breed effect on liveweight gain, after slaughter the superior traits of the crossbreeds were revealed. Carcasses of CH steers were 32 kg heavier than those of DH steers, and when the rearing period was prolonged from 21 to 28 months of age the difference was even greater, where carcasses from CL steers weighed 50 kg more than carcasses from DL steers. Hence, as stated in previous studies [39], comparison of liveweight gain is not sufficient when evaluating the effects of beef breed crosses. The heavier carcass of the C steers was accompanied by a higher dressing percentage and a higher conformation score. The breed difference in conformation was greater for the low-intensity steers slaughtered at a higher age and weight (L) than for the more intensively fed steers slaughtered at a lower age and weight (H), as the C steers increased their conformation score from the early to the later slaughter age (CH to CL), whereas the conformation score of the D steers remained the same (DH and DL). Note that the results from the C steers should be considered with caution, as all of these steers were descendent from a single Charolais sire, the dominant sire on the market at that time, from the breeding company characterized with superior breeding index for carcass gain (121, where 100 is average), but rather low for conformation (91) and fatness (94) [19].

As there are differences in the proportions of muscle and fat deposited in cattle of different breeds, the higher conformation score of the C steers was reflected in a greater proportion of valuable retail cuts, and a smaller proportion of bone, compared to the D steers. Hence, the greater muscle proportions in dairy × beef crossbreeds than in pure dairy breeds give a more valuable carcass, expressed as kg saleable meat. On the other hand, dairy breeds in general deposit a higher proportion of fat than late-maturing beef breeds, which in our steers was reflected in a tendency for higher scores of visually estimated marbling, intramuscular fat, in the *M. longissimus dorsi* in the D steers than in the C steers. This intramuscular fat might explain the tendency for lower feed efficiency in the DL steers compared to the CL steers during the last indoor period [40]. More marbling might also result in higher and less variable eating quality in the dairy steers, with more tender and juicy meat [41]. Previous studies have reported carcasses with leaner meat and less fat in dairy × beef breeds compared to pure dairy breeds, when fed on similar feed rations and slaughtered at a similar liveweight (e.g., References [14,37,39,42]). However, despite numerical differences, no statistically significant effect of breed on the carcass fatness score or proportion of trim fat was found in the present study. This result was surprising, as lipogenic activity was higher in subcutaneous deposits than in intramuscular fat deposits [43], which meant that fat was preferentially stored subcutaneously rather than intramuscularly. On the other hand, there was sizable individual within-breed variation in both fatness score and amount of trim fat (Table 5).

In the present study, 97% (all except one that was too lean) of the C steers reached carcass weights (275 to 424.9 kg), conformation scores (at least O−), and fatness scores (2 to 4−) qualifying them for premium prices on the current Swedish market [44]. The corresponding value for the D steers was 59%, where the main problem was insufficient conformation, sometimes combined with a very low carcass weight. Thus, the D steers would have benefited from a more extended rearing period; especially the group slaughtered at 21 months of age.

Increased indoor feed intensity resulted in higher dietary intake and NDF intake expressed as kg day^−1^ during the indoor period 1 (12% and 16%, respectively) and indoor period 2 (23% and 8%, respectively). The difference during indoor period 1 in dietary intake between H steers and L steers slaughtered was due to the spring-born H steers being older and larger during that period than the autumn-born L steers. Hence, when expressed as a proportion of liveweight, we found no differences in feed intake. Differences in dietary intake, liveweight gain, and feed efficiency during indoor period 2 were mainly due to the composition of the different grass-clover silages fed. The aim of feeding late-cut silage to the autumn-born low-intensity steers was to restrain feed intake and weight gain so that they could compensate for these during the subsequent grazing period.

Interestingly, H steers slaughtered at lower weight and age had a numerically higher average fatness score than L steers slaughtered at a higher weight and age. This effect was most likely a result of the H steers slaughtered at low age, having a longer indoor finishing period before slaughter (average 163 days) than the L steers (average 100 days), combined with higher weight gain during indoor periods than on grass. The finding supports the assumption that the length of the last indoor period showed a positive relationship with the fatness score, which increased by 0.4 units per month.

The proportion of animal lifetime weight gain obtained from pasture was 13% for H steers and 30% for L steers. These figures may also seem low for nemoral and boreal climate zones but are less important than the proportion of income from pastures and carcasses. Use of pastures categorized as “keeping general biological values” [7] and the stocking rate of 0.44 ha animal^−1^ used for the spring-born moderately high-intensity steers meant that the system qualified for an environmental payment corresponding to 4% of the economic value of the carcasses, besides the single-farm payment for which all systems are qualified. Lowering the indoor feed intensity to the level of the autumn-born low-intensity steers and allowing them to graze another summer increased the agri-environmental payment per steer, which was based on 1.32 ha animal^−1^. Thus, the use of pastures categorized as “keeping general biological values” for the autumn-born low-intensity steers yielded an agri-environmental payment corresponding to 10% of the carcass value. If pastures categorized as “keeping specific biological values” [7] had been used for the autumn-born low-intensity steers, the agri-environmental payment would have increased further, to 28% of carcass value.

In contrast to indoor feeds, herbage from semi-natural pastures usually has a negative cost when the agri-environmental payment is included in the production budget [45]. As the agri-environmental payment is obtained per hectare of grassland [7], land with low biomass production could be more attractive to farmers as every animal can manage a greater area. Utilized herbage mass in the semi-natural pastures at the experimental site, calculated based on animal weight gain and stocking rate, was relatively high, 1.6 tonnes ha^−1^. Since the requirement for obtaining the agri-environmental payment is a well-grazed area, animals on poorly productive grazing earn a larger payment per head. Herbage biomass from semi-natural pastures, and also the agri-environmental payment that can be obtained per animal, may differ by as much as six fold [46]. Spörndly and Glimskär [47] recently showed that the general animal stocking rate on Swedish semi-natural pastures is higher than previously estimated [48]. This result might be due to either an actual increase in biomass production as a result of increased levels of atmospheric nitrogen and/or a longer vegetation period or to different ways of estimating biomass production. If biomass production is higher, beef production under present conditions receives a lower income from the agri-environmental payment per animal than previously anticipated.

In general, liveweight gain was lower during the grazing periods than during the indoor periods (Table 4). The energy concentration in herbage on semi-natural pastures is often low, and this is particularly true for *Deschampsia* cespitosa [49], the grass species dominating the pasture in this study. However, the concentrations of metabolizable energy and NDF were roughly similar in the pasture herbage and the grass-clover silages fed (Table 1, Figure 2). Quantity, and not quality, was probably the limiting factor for weight gain in the present study, especially during grazing period 1. The average sward height on the pastures was 4.3 and 4.9 cm during grazing periods 1 and 2, respectively. In an earlier study of dairy steers on semi-natural pastures, low sward height, below 6 cm, has been shown to restrict weight gain [50]. Similar results have been reported in earlier studies of growing cattle grazing the same pastures as in the present study [51,52]. Although weight gain on pasture was most likely restricted by insufficient herbage mass, decreasing the stocking rate would not improve the production system as a whole since short sward height at the end of the grazing period is required to obtain the agri-environmental payment for semi-natural pastures [7]. Although the final sward height was unnecessarily low at the end of grazing period 1 (3 cm), the optimal sward height on semi-natural pastures, in general, was a trade-off between beef production and nature conservation aspects.

When grazing permanent pastures such as the semi-natural grasslands, infection with pasture-borne gastrointestinal nematodes such as *Ostertagia ostertagi* in naïve grazers is inevitable. This study found that half the steers of both breeds were infected with gastrointestinal nematodes during their first grazing season. Both breeds showed lower weight gain, but there were indications that the impact of parasite infection was more severe in C steers than in D steers [21]. This effect may be due to dairy × beef crossbreeds allocating a higher proportion of nutrition to weight gain and a lower proportion to the immune system compared to purebred dairy calves [53]. Over time, beef cattle breeds have been exposed to lower selection pressure for resistance to gastrointestinal nematode infection than their dairy counterparts [54], outweighing a possible heterosis effect on resistance from crossbreeding [55]. By their second grazing period, the steers in this study had developed immunity to the parasites. Although there was no significant difference in liveweight gain during this grazing period, the weight gain in the C steers was numerically almost 50% higher than in the D steers, indicating the superior potential of C steers for weight gain when parasite infection was not suppressing growth. Across breeds, there was no overall effect of parasite infection on the total liveweight gain from weaning to slaughter, indicating that the infected steers compensated in terms of weight gain during the remainder of the rearing period.

From a nature conservation perspective, it is beneficial to decrease the feed intensity in beef production by a change from intensive indoor grain-based rearing to extensive rearing utilizing biodiverse semi-natural pastures [1]. However, this environmental goal may conflict with another, reducing greenhouse gas emissions. All ruminant production results in enteric emissions of the greenhouse gas methane [56], although dairy × beef crossbreeds from dairy cows represent the least bad alternative in this regard [13]. Lowering the intensity of beef production systems, and thereby prolonging the rearing period, increases the emissions per kg of edible meat [13]. There is an ongoing discussion on the ability of semi-natural pastures to sequester atmospheric carbon and thereby counterbalance some enteric emissions of methane, but the scope is most likely limited [57]. No matter what the environmental issue is in focus, the present study demonstrated that high carcass quality and preservation of semi-natural pastures could be achieved successfully within the same beef production system.

## 5. Conclusions

The use of beef breed semen, rather than dairy breed semen on dairy cows, did not affect liveweight gain in the offspring in this study. However, dairy × beef breed crosses had higher carcass weight with a higher conformation score and a higher proportion of valuable retail cuts, all traits of economic value than purebred dairy steers. The breed difference in conformation was greater for extensively raised steers slaughtered at a higher age and weight than for more intensively raised steers slaughtered at a lower age and weight. Hence, replacing purebred dairy steers with dairy × beef crosses in production based on forage and semi-natural pastures results in more commercially valuable carcasses.

## Figures and Tables

**Table 1:** Proportion (% of dry matter) of grass-clover silage, rolled barley, rolled peas, and expro meal in the total mixed ration fed to young purebred dairy and cross-bred dairy × beef steers at different liveweights during their first indoor period.

Liveweight, kg	Silage	Barley	Peas	Expro Meal ^a^
−125	58	14	14	14
125–175	76	8	8	8
175–225	92	8	0	0
225–275	95	5	0	0
275–	100	0	0	0

^a^ Protein feed (heat-treated rapeseed meal).

**Table 2:** Chemical composition of rolled barley, rolled peas, and expro meal (*n* = 1) fed to young purebred dairy and cross-bred dairy × beef steers during their first indoor period (s.d. = standard deviation, DM = dry matter, ME = metabolizable energy, CP = crude protein, NDF = neutral detergent fiber).

Component, kg^−1^ DM	Barley	Peas	Expro Meal ^a^
DM, g kg^−1^	868	863	888
ME, MJ	13.4	14.2	12.4
CP, g	95	206	330
NDF, g	180	129	310
Starch, g	522	479	70
Crude fat, g	27	20	39
Ash, g	21	25	68

^a^ Protein feed (heat-treated rapeseed meal).

**Table 3:** Chemical composition of grass-clover silage fed during indoor period (IP) 1, 2, and 3 to purebred dairy and cross-bred dairy × beef steers in two different production systems with varying birth season and moderately high (H) or low (L) indoor feed intensity (s.d. = standard deviation, DM = dry matter, ME = metabolizable energy, CP = crude protein, NDF = neutral detergent fiber, NH_4_-N = ammonium nitrogen).

Component, kg^−1^ DM	IP1			IP2						IP3		
	H and L			H			L			L		
	Mean	s.d.	*n*	Mean	s.d.	*n*	Mean	s.d.	*n*	Mean	s.d.	*n*
DM, g kg^−1^	350	62	41	379	104	28	473	111	31	460	34	21
ME, MJ	10.7	0.5	10	10.2	0.6	6	8.5	0.1	6	10.5	0.3	4
CP, g	116	7	10	122	19	6	75	11	6	109	6	4
NDF, g	563	28	10	546	44	6	618	11	6	561	41	4
Ash, g	82	3	10	93	7	6	61	2	6	76	5	4
pH	4.4	0.4	3	4.7	0.5	3	5.1	0.5	2	4.7	0.2	2
NH_4_-N, g ^a^	1.94	0.47	3	6.76	1.88	3	3.61	0.75	2	1.24	0.30	2

^a^ Including N from the silage additive.

**Table 4:** Daily feed intake, daily liveweight gain, and feed efficiency (ME = metabolizable energy) of purebred dairy (D) and cross-bred dairy × beef (C) steers in two production systems (Prod.sys), where spring-born animals were kept on a moderately high feed intensity and then slaughtered at 21 months (mo) of age (H), while autumn-born animals were kept on a low feed intensity and then slaughtered at 28 months of age (L). Least square mean, pooled standard error of the mean (s.e.), and significance of the main effects of breed and production system.

Item	Breed		Prod.sys.		s.e.	Level of Significance	
*n*	D	C	H	L		Breed	Prod.sys.
	29	32	30	31			
**Indoor period 1**							
Initial liveweight, kg	115.3	140.1	121.5	133.9	7.78	0.0514	ns
Dietary intake, kg of dry matter	5.43	5.63	5.85	5.21	0.12	0.0356	0.0256
Dietary intake, % of liveweight	3.13	2.50	2.60	3.02	0.25	ns	ns
Intake of neutral detergent fiber, kg	2.75	2.95	3.06	2.64	0.09	0.0045	0.0308
Liveweight gain, kg day^−1^	1.05	1.06	1.08	1.02	0.03	ns	ns
Feed efficiency, ME MJ g gain^−1^	58.7	59.4	60.1	58.1	0.78	ns	0.0946
**Grazing period 1**							
Initial liveweight, kg	304.5	331.0	372.4	263.1	0.95	0.0075	<0.0001
Liveweight gain, kg day^−1^	0.51	0.55	0.46	0.60	0.03	ns	0.0050
**Indoor period 2**							
Initial liveweight, kg	377.4	409.7	438.3	348.9	6.80	0.0010	<0.0001
Dietary intake, kg of dry matter	10.5	11.0	11.8	9.6	0.24	ns	0.0037
Dietary intake, % of liveweight	2.30	1.97	2.04	2.23	0.17	ns	ns
Intake of neutral detergent fiber, kg	5.98	6.47	6.47	5.98	0.13	0.0204	0.0212
Liveweight gain, kg day^−1^	0.89	0.94	1.14	0.68	0.03	ns	<0.0001
Feed efficiency, ME MJ g gain^−1^	114.4	116.1	108.4	122.1	2.52	ns	0.0218
**Grazing period 2**							
Initial liveweight, kg	466.4	517.0	-	491.7	12.82	0.0196	-
Liveweight gain, kg day^−1^	0.53	0.73	-	-	0.13	ns	-
**Indoor period 3**							
Initial liveweight, kg	548.2	602.3	-	575.2	12.50	0.0061	-
Dietary intake, kg of dry matter	13.5	14.3	-	13.9	0.20	0.0578	-
Dietary intake, % of liveweight	2.17	2.08	-	2.16	0.03	ns	-
Intake of neutral detergent fiber, kg	7.45	7.82	-	7.64	0.10	0.0658	-
Liveweight gain, kg day^−1^	1.21	1.34	-	1.28	0.11	ns	-
Feed efficiency, ME MJ g gain^−1^	127.2	122.0	-	124.6	15.00	0.0578	-
**From weaning to slaughter**							
Liveweight gain, kg day^−1^	0.84	0.86	0.94	0.77	0.02	ns	<0.0001

**Table 5:** Carcass characteristics of purebred dairy (D) and cross-bred dairy × beef (C) steers in two production systems (Prod.sys), where spring-born animals were kept on a moderately high feed intensity and then slaughtered at 21 months (mo) of age (H), while autumn-born animals were kept on a low feed intensity and then slaughtered at 28 months of age (L). Least square mean, pooled standard error of the mean (s.e.), and significance of the main effects of breed and production system.

Item	Breed		Prod.sys.		s.e.	Level of Significance	
*n*	D	C	H	L		Breed	Prod.sys.
	29	32	30	31			
Slaughter							
Liveweight, kg	640	682	628	695	10.4	0.0026	0.0002
Carcass weight, kg	294	335	299	329	4.9	<0.0001	0.0002
Dressing, %	45.8	49.1	47.6	47.3	0.36	0.0001	ns
Conformation ^a^	4.0	5.7	4.6	5.1	0.20	0.0001	0.0844
Fatness ^b^	7.2	7.4	7.9	6.8	0.24	ns	ns
Marbling ^c^	2.0	1.5	1.7	1.8	0.17	0.0540	ns
**Cutting up**							
HQ ^d^, kg	74.6	85.7	77.3	83.1	1.21	<0.0001	0.0025
Retail cuts^e^, % of HQ	36.7	38.3	37.1	37.9	0.31	0.0040	0.0859
Grade 2 meat ass.^f^, % of HQ	21.0	22.2	19.9	23.2	0.32	0.0004	<0.0001
Grade 3 meat ass.^g^, % of HQ	9.7	9.3	11.6	7.3	0.44	ns	<0.0001
Trim fat, % of HQ	7.8	6.9	7.0	7.7	0.36	ns	ns
Bone, % of HQ	21.6	20.2	21.0	20.7	0.24	0.0021	ns

^a^ EUROP system: 4 = O−, 5 = O, 6 = O+. Interaction Breed × Prod.sys. *p* = 0.0105. ^b^ EUROP system: 6 = 2+, 7 = 3−, 8 = 3. ^c^ Visually determined in *Musculus longissimus dorsi* between the 10th and 11th ribs on a scale 1 = lean and 5 = well-marbled. ^d^ Right hindquarter. ^e^ High-value retail cuts; strip loin, fillet, topside, outside round, eye of round, top rump, and rump steak. ^f^ Commercial meat cuts estimated to contain 10% fat. ^g^ Commercial meat cuts estimated to contain 23% fat.

## Supplementary Materials

The following are available online at https://www.mdpi.com/2076-2615/9/12/1064/s1, Table S1: Group data on purebred dairy and dairy × beef steers in forage and pasture-based production systems, where spring-born cattle were kept on a moderately high indoor feed intensity and slaughtered at 21 months of age, while autumn-born cattle were kept on a low indoor feed intensity and slaughtered at 28 months of age. (IP = indoor period), Table S2: Individual data on purebred dairy and dairy × beef steers in forage and pasture-based production systems, where spring-born cattle were kept on a moderately high feed intensity and slaughtered at 21 months of age, while autumn-born cattle were kept on a low feed intensity and slaughtered at 28 months of age (IP = indoor period, GP = grazing period).

## References

[1] Emanuelsson U. (2009). The Rural Landscapes of Europe—How Man Has Shaped European Nature.

[2] Luoto M., Rekolainen S., Aakkula J., Pykalä J. (2003). Loss of plant species richness and habitat connectivity in grasslands associated with agricultural change in Finland. AMBIO.

[3] Auffret A.G., Kimberley A., Plue J., Waldén E. (2018). Super-regional land-use change and effects on the grassland specialist flora. Nat. Commun..

[4] Godfray H.C.J., Beddington J.R., Crute I.R., Haddad L., Lawrence D., Muir J.F., Pretty J., Robinson S., Thomas S.M., Toulmin C. (2010). Food security: The challenge of feeding 9 billion people. Science.

[5] World Wildlife Fund (2012). WWF Baltic Ecoregion Programme—Sorting out the Goods. Agri-Environment Measures in the Baltic Sea Member States. http://wwf.panda.org/knowledge_hub/where_we_work/baltic/publications/.

[6] Blumentrath C., Stokstad G., Dramstad W., Eiter S. (2014). Agri-Environmental Policies and Their Effectiveness in Norway, Austria, Bavaria, France, Switzerland and Wales: Review and Recommendations. Skog OG Landskap 11. https://brage.bibsys.no/xmlui/handle/11250/2440142.

[7] Swedish Board of Agriculture (2018). Pastures and Meadows. www.jordbruksverket.se/amnesomraden/stod/jordbrukarstod/stodochersattningar2018/miljoersattningar/betesmarkerochslatterangar.4.6c64aa881525004b53bda366.html.

[8] Hessle A., Kumm K.-I. (2011). Use of beef steers for profitable management of biologically valuable semi-natural pastures in Sweden. J. Nat. Conserv..

[9] Salevid P., Kumm K.-I. (2011). Searching for economically sustainable Swedish beef production systems based on suckler cows after decoupling EU income support. Outlook Agric..

[10] (2017). Gård & Djurhälsan. Carcass Quality Outcome for Whole Year. https://www.gardochdjurhalsan.se/sv/not/kunskapsbank/statistik/slaktstatistik/.

[11] (2018). Växa Sverige. Cattle Statistics. https://www.gardochdjurhalsan.se/sv/not/kunskapsbank/statistik/kap-statistik/.

[12] Swedish Dairy Association (2013). Cattle Statistics. https://www.gardochdjurhalsan.se/sv/not/kunskapsbank/statistik/kap-statistik/.

[13] De Vries M., Van Middelaar C.E., De Boer I.J.M. (2015). Comparing environmental impacts of beef production systems: A review of life cycle assessments. Livest. Sci..

[14] Jukna V., Jukna Č., Pečiulaitienė N. (2009). The beef production efficiency of milk cattle used crossed with different intensive beef cattle breeds. Biotech. Anim. Husb..

[15] Huuskonen A., Pesonen M., Kamarainen H., Kauppinen R. (2014). Production and carcass traits of purebred Nordic Red and Nordic Redxbeef breed crossbred bulls. J. Agric. Sci..

[16] Vestergaard M., Jørgensen K.F., Çakmakçı C., Kargo M., Therkildsen M., Munk A., Kristensen T. (2019). Performance and carcass quality of crossbred beef × Holstein bull and heifer calves in comparison with purebred Holstein bull calves slaughtered at 17 months of age in an organic production system. Livest. Sci..

[17] Eriksson S., Gullstrand P., Fikse W.F., Jonsson E., Stålhammar H., Wallenbeck A., Hessle A. Crossbreeding with beef bulls in Swedish dairy herds-analysis of calving and carcass traits. Proceedings of the 69th Annual Meeting of the European Federation of Animal Science.

[18] Webster A.J.F. (1989). Bioenergetics, bioengineering and growth. Anim. Sci..

[19] VikingGenetics Charolais. http://www.vikinggenetics.se/kottraser/charolais/tillgangliga-tjurar.

[20] Spörndly R. (2003). Feed Tables for Ruminants 2003.

[21] Höglund J., Hessle A., Zaralis K., Arvidsson-Segerkvist K., Athanasiadou S. (2018). Weight gain and resistance to gastrointestinal nematode infections in two genetically diverse groups of cattle. Vet. Par..

[22] Frame J., Davis A., Baker R.D., Grant S.A., Laidlaw A.S. (1993). Herbage mass. Sward Measurement Handbook.

[23] Dumas J.B.A. (1831). Procedes de l’Analyse Organique. Ann. Chim. Phys..

[24] Chai W., Udén P. (1998). An alternative oven method combined with different detergent strengths in the analysis. Anim. Feed Sci. Technol..

[25] Lindgren E. (1979). The Nutritional Value of Roughages Determined In Vivo and by Laboratory Methods.

[26] Axelsson J. (1941). Der Gehalt des Futters an umsetzbarer Energie. Züchtungskunde.

[27] Åman P., Hesselman K. (1984). Analysis of starch and other main constituents of cereal grains. Swed. J. Agric. Res..

[28] EU Council Directive Directive 1998/64/EC. Official Journal of the European Communities L257/14. Commission of the European Communities, Brussels. https://eur-lex.europa.eu/homepage.html.

[29] Andersson R., Hedlund B. (1983). HPLC analysis of organic acids in lactic acid fermented vegetables. Z. Lebensm. Unters. Forsch..

[30] Council of the European Union Council Regulation (EC) No 1234/2007. https://eur-lex.europa.eu/homepage.html.

[31] Commission of the European Union Commission Regulation (EC) No 1249/2008. https://eur-lex.europa.eu/homepage.html.

[32] (1998). SJVFS 127. Directions of Classifications of Carcasses from the Swedish Board of Agriculture. Swedish Board of Agriculture. https://www.jordbruksverket.se/forfattningar/forfattningssamling/forfattningar19932017medseparataandringsforskrifterochbilagor.4.160b021b1235b6bb8618000633.html.

[33] USDA Official USDA Marbling Photographs. https://www.dmsfulfillment.com/NCBA/Secure/StoreItem.aspx?ID=16313&ITEMS=CATALOG&CAT=165&TP=180.

[34] SAS Institute Inc (2018). SAS Version 9.4.

[35] Kenward M.G., Roger J.H. (1997). Small sample iference for fixed effects from restricted maximum likelihood. Biometrics.

[36] Pogorzelska-Przybyłek P., Nogalski Z., Sobczuk-Szul M., Purwin C., Momot M. (2018). Carcass characteristics of grass-fed crossbred bulls and steers slaughtered at two different ages. Can. J. Anim. Sci..

[37] Keane M.G. (2010). A comparison of finishing strategies to fixed slaughter weights for Holstein Friesian and Belgian Blue × Holstein Friesian steers. IRISH J. Agric. Food Res..

[38] Hessle A., Nadeau E., Johnsson S. (2007). Finishing of dairy steers having grazed semi-natural grasslands. Livest. Sci..

[39] Forrest R.J. (1977). A comparison of birth growth and carcass characteristics between Holstein-Frisian steers and Charolais × Holstein (F1) crossbreds. Can. J. Anim..

[40] Lawrence T., Fowler V., Novakofski J. (2015). Growth of Farm Animals.

[41] Corbin C.H., O’Quinna T.G., Garmyn A.J., Legako J.K., Hunt M.-R., Dinh T.T.N., Rathmann R.J., Brooks J.C., Miller M.F. (2015). Sensory evaluation of tender beef strip loin steaks of varying marbling levels and quality treatments. Meat Sci..

[42] McGee M., Keane M.G., Neilan R., Moloney A.P., Caffrey P.J. (2005). Production and carcass traits of high dairy genetic merit Holstein, standard dairy genetic merit Friesian and Charolais × Holstein-Friesian male cattle. IRISH J. Agric. Food Res..

[43] Mendizabal J.A., Alberti P., Eguinoa P., Arana A., Soret B., Purroy A. (1999). Adipocyte size and lipogenic enzyme activities in different adipose tissue depots in steers of local Spanish breeds. Anim. Sci..

[44] HK Scan Agri HK Scan Agri Notering. http://www.hkscanagri.se/notering/.

[45] Kumm K.-I. (2009). Produktionskostnad för Grovfoder Till Köttdjur.

[46] Heinsoo K., Melts I., Sammul M., Holm B. (2010). The potential of Estonian semi-natural grasslands for bioenergy production. Agric. Ecosyst. Environ..

[47] Spörndly E., Glimskär A. (2018). Grazing Livestock and Stocking Rate in Swedish Semi-Natural Pastures.

[48] Steen E., Matzon C., Svensson C. (1972). Landscape Management with Grazing Livestock.

[49] Andersson A. (1999). Nutritional Value in Grasses from Semi-Natural Pastures. Master Thesis.

[50] Spörndly E., Olsson I., Burstedt E. (2000). Grazing by steers at different sward surface heights on extensive pastures: A study of weight gain and fat deposition. Acta Agric. Scand. Sect. A Anim. Sci..

[51] Hessle A., Nadeau E., Johnsson S. (2007). Beef heifer production as affected by indoor feed intensity and slaughter age when grazing semi-natural grasslands in summer. Livest. Sci..

[52] Hessle A., Dahlström F., Wallin K. (2011). Alternative production systems for male Charolais cross-bred cattle using semi-natural grasslands. Acta Agric. Scand. Sect. A Anim. Sci..

[53] Rauw W.M., Kanis E., Noordhuizen-Stassen E.N., Grommers F.J. (1998). Undesirable side effects of selection for high production efficiency in farm animals: A review. Livest. Prod. Sci..

[54] Dahlström A. (2006). Pastures, Livestock and Stocking Rate 1620–1850. Nature Conservation Aspects on Historical Grazing in Southern and Central Sweden. Ph.D. Thesis.

[55] Williams J.L., Aguilar I., Rekaya R., Bertrand J.K. (2010). Estimation of breed and heterosis effects for growth and carcass traits in cattle using published crossbreeding studies. J. Anim. Sci..

[56] Poore J., Nemecek T. (2018). Reducing food’s environmental impacts through producers and consumers. Science.

[57] Garnett T., Godde C., Muller A., Röös E., Smith P., De Boer I., Zu Ermgassen E., Herrero M., Van Middelaar C., Schader C. (2017). Grazed and Confused? Food Climate Research Network. https://fcrn.org.uk/fcrn-publications/reports.

