# Supplementary material for: Beef Production Systems with Steers of Dairy and Dairy × Beef Breeds Based on Forage and Semi-Natural Pastures
Source: Animals (Basel). 2019 Dec 2;9(12):1064. doi: 10.3390/ani9121064 (PMC6940778; doi:10.3390/ani9121064)
Supplement: Supplementary file 1 [file animals-09-01064-s001.pdf]

Supplementary Data

Table S1. Group data on purebred dairy and dairy x beef steers in forage and pasture-based production systems, where spring-born cattle were kept on a moderately high indoor feed intensity and slaughtered at 21 months of age, while autumn-born cattle were kept on a low indoor feed intensity and slaughtered at 28 months of age. (IP = indoor period)

Pen. no	13	14	15	16	17	18	19	20
Breed[a]	1	2	1	2	1	2	1	2
Production system[b]	1	1	1	1	1	1	1	1
Pen pair, no.	1	1	2	2	3	3	4	4
Parasites[c]	1	1	2	2	1	1	2	2
Dietary intake, kg of dry matter IP1	6.323	5.812	6.245	6.134	5.745	5.837	5.489	5.380
Dietary intake, % of liveweight IP1	2.357	2.629	2.481	2.542	2.429	2.534	2.485	3.003
Intake of neutral detergent fiber, kg IP1	3.427	3.037	3.358	3.248	3.040	3.046	2.843	2.673
Feed efficiency, ME MJ g gain^{-1} IP1	62.88	62.98	65.19	60.88	59.67	56.91	56.79	58.30
Dietary intake, kg of dry matter IP2	11.021	11.308	12.124	12.111	12.372	12.133	11.638	11.472
Dietary intake, % of liveweight IP2	0.091	2.211	2.297	2.346	2.233	2.166	2.252	2.428
Intake of neutral detergent fiber, kg IP2	7.047	5.969	6.491	6.485	6.724	6.526	6.348	6.261
Feed efficiency, ME MJ g gain^{-1} IP2	114.66	113.50	117.57	110.69	103.94	101.99	103.33	103.08
Dietary intake, kg of dry matter IP3	.	.	.	.	.	.	.	.
Dietary intake, % of liveweight IP3	.	.	.	.	.	.	.	.
Intake of neutral detergent fiber, kg IP3	.	.	.	.	.	.	.	.
Feed efficiency, ME MJ g gain^{-1} IP3	.	.	.	.	.	.	.	.

[a]1 is dairy x beef crossbreed, 2 is dairy breed.
[b]1 is spring-born, moderately high indoor feed intensity with 21 months slaughter age, 2 is autumn-born, low indoor feed intensity and 28 months slaughter age.
[c]1 is not infected, 2 is infected.

Table S1 cont.

Pen. no	21	22	23	24	25	26	27	28
Breed[a]	1	2	1	2	1	2	1	2
Production system[b]	2	2	2	2	2	2	2	2
Pen pair, no.	5	5	6	6	7	7	8	8
Parasites[c]	2	2	2	2	1	1	1	1
Dietary intake, kg of dry matter IP1	5.522	5.161	4.881	4.928	5.445	5.131	5.295	5.158
Dietary intake, % of liveweight IP1	2.446	2.629	2.619	2.991	2.588	2.696	2.833	5.731
Intake of neutral detergent fiber, kg IP1	2.898	2.613	2.428	2.376	2.838	2.592	2.696	2.531
Feed efficiency, ME MJ g gain^{-1} IP1	61.91	62.62	56.07	57.51	54.93	60.21	56.06	52.36
Dietary intake, kg of dry matter IP2	10.619	9.174	9.165	9.456	10.995	8.659	9.850	9.339
Dietary intake, % of liveweight IP2	2.255	2.245	2.159	2.298	2.363	2.094	2.286	2.433
Intake of neutral detergent fiber, kg IP2	6.568	5.674	5.669	5.849	6.801	5.355	6.092	5.776
Feed efficiency, ME MJ g gain^{-1} IP2	115.75	124.67	118.33	119.35	133.61	117.25	120.09	126.10
Dietary intake, kg of dry matter IP3	14.429	13.649	13.829	13.741	14.886	13.502	13.612	13.358
Dietary intake, % of liveweight IP3	2.188	2.258	2.070	2.218	2.083	2.100	1.945	2.145
Intake of neutral detergent fiber, kg IP3	7.820	7.398	7.518	7.469	8.190	7.434	7.699	7.587
Feed efficiency, ME MJ g gain^{-1} IP3	89.74	111.30	94.29	100.27	134.30	112.38	185.80	168.69

[a]1 is dairy x beef crossbreed, 2 is dairy breed.

[b]1 is spring-born, moderately high indoor feed intensity with 21 months slaughter age, 2 is autumn-born, low indoor feed intensity and 28 months slaughter age.

[c]1 is not infected, 2 is infected.

Table S2. Individual data on purebred dairy and dairy x beef steers in forage and pasture-based production systems, where spring-born cattle were kept on a moderately high feed intensity and slaughtered at 21 months of age, while autumn-born cattle were kept on a low feed intensity and slaughtered at 28 months of age (IP = indoor period, GP = grazing period)

Animal ID	8247	8255	8271	8286	8244	8254	8276	8294
Date of birth	150418	150425	150505	150509	150415	150425	150506	150511
Pen no.	13	13	13	13	14	14	14	14
Breed[a]	1	1	1	1	2	2	2	2
Production system[b]	1	1	1	1	1	1	1	1
Pair of calves, no	1	2	3	4	1	2	3	4
Parasites[c]	1	1	1	1	1	1	1	1
Weight gain IP1, kg d^{-1}	1.210	1.072	1.115	0.991	0.987	0.982	1.045	1.066
Weight gain GP1, kg d^{-1}	0.243	0.560	0.577	0.835	0.412	0.500	0.539	0.518
Weight gain IP2, kg d^{-1}	1.175	1.349	1.198	1.127	1.063	1.012	0.944	1.123
Weight gain GP2, kg d^{-1}	.	.	.	.	.	.	.	.
Weight gain IP3, kg d^{-1}	.	.	.	.	.	.	.	.
Weight gain total, kg d^{-1}	0.951	1.003	0.995	0.982	0.855	0.864	0.890	0.937
Weight at slaughter, kg	676.5	680.5	675.0	654.0	580.0	578.5	564.0	601.0
Carcass weight, kg	329.8	333.7	333.7	320.5	262.6	271.1	253.2	267.5
Dressing, %	48.8	49.0	49.4	49.0	45.3	46.9	44.9	44.5
Conformation, score[d]	5	6	5	6	3	4	4	4
Fatness, score[e]	7	6	8	7	6	7	6	6
Marbling, score[f]	2	1	2	1	3	1	2	1
Weight of HQ[g], kg	84	84.5	84.5	81.5	68.5	69.5	65.5	69
Trim fat, % of HQ	4.87	4.39	7.88	4.61	7.99	6.81	6.09	5.78
Gr. 2 meat ass.[h], % of HQ	22.1	23.1	20.6	22.2	20.2	20.0	20.6	21.6
Gr. 3 meat ass.[i], % of HQ	11.8	10.2	12.4	12.0	9.8	9.6	11.2	11.4
Bone, % of HQ	20.8	20.2	19.1	19.9	21.4	22.0	21.7	21.6
Retail cuts[j], % of HQ	37.2	38.8	35.3	37.8	36.4	37.8	36.6	35.2
Slaughter age, d	648	641	631	627	651	641	630	625
Relative age[k], d	3	10	20	24	0	10	21	26
Initial weight IP1, kg	157	132.5	131.5	117.5	112.5	106.5	77.5	89
Initial weight GP1, kg	494	431	442	393.5	387.5	380	368.5	386
Initial weight IP2, kg	528.5	510.5	524	512	446	451	445	459.5
Initial weight GP2, kg	.	.	.	.	.	.	.	.
Initial weight IP3, kg	.	.	.	.	.	.	.	.

[a]1 is dairy x beef crossbreed, 2 is dairy breed.

[b]1 is spring-born, moderately high indoor feed intensity with 21 months slaughter age, 2 is autumn-born, low indoor feed intensity and 28 months slaughter age.

[c]1 is not infected, 2 is infected.

[d]EUROP system on a scale 1 = poor, 15 = excellent.

[e]EUROP system on a scale 1 = lean, 15 = fat.

[f]Visually determined in *Musculus longissimus dorsi* between 10th and 11th ribs on a scale 1 = lean and 5 = well marbled.

[g]Right hind quarter.

[h]Commercial cut meat assortment estimated to contain 10% fat.

[i]Commercial cut meat assortment estimated to contain 23% fat.

[j]High-value retail cuts; strip loin, fillet, topside, outside round, eye of round, top rump, and rump steak

[k]Relative to the oldest animal in that production system group.

Table S2 cont.

Animal ID	8299	8301	8326	8332	8296	8298	8321	8329
Date of birth	150514	150515	150601	150603	150513	150515	150526	150602
Pen no.	15	15	15	15	16	16	16	16
Breed[a]	1	1	1	1	2	2	2	2
Production system[b]	1	1	1	1	1	1	1	1
Pair of calves, no	5	6	7	8	5	6	7	8
Parasites[c]	2	2	2	2	2	2	2	2
Weight gain IP1, kg d^{-1}	1.016	1.079	1.002	1.110	1.178	1.164	1.081	1.027
Weight gain GP1, kg d^{-1}	0.243	0.194	0.444	0.581	0.180	0.377	0.243	0.412
Weight gain IP2, kg d^{-1}	1.230	1.140	0.988	0.910	1.320	1.099	0.991	1.118
Weight gain GP2, kg d^{-1}	.	.	.	.	.	.	.	.
Weight gain IP3, kg d^{-1}	.	.	.	.	.	.	.	.
Weight gain total, kg d^{-1}	0.873	0.858	0.847	0.906	0.951	0.931	0.827	0.889
Weight at slaughter, kg	614.0	631.0	581.0	636.0	650.0	634.5	573.0	574.0
Carcass weight, kg	302.8	307.2	291.1	310.2	300.9	296.5	264.1	271.5
Dressing, %	49.3	48.7	50.1	48.8	46.3	46.7	46.1	47.3
Conformation, score[d]	4	4	5	4	4	4	4	4
Fatness, score[e]	8	8	9	9	8	9	9	8
Marbling, score[f]	2	1	1	1	2	1	2	1
Weight of HQ[g], kg	77.5	77.5	76	79.5	76	73.5	68.5	72
Trim fat, % of HQ	5.47	5.31	5.95	6.95	7.42	11.19	7.59	5.60
Gr. 2 meat ass.[h], % of HQ	20.4	21.1	20.2	19.9	20.0	19.6	19.8	20.0
Gr. 3 meat ass.[i], % of HQ	10.7	11.0	11.4	11.8	11.2	11.0	10.9	11.8
Bone, % of HQ	20.4	21.3	19.3	19.5	21.1	19.7	23.3	21.5
Retail cuts[j], % of HQ	39.7	38.1	38.9	38.7	36.3	35.2	35.6	37.9
Slaughter age, d	657	656	639	637	658	656	645	638
Relative age[k], d	29	30	47	49	28	30	41	48
Initial weight IP1, kg	155.5	180	136	160	150.5	145.5	139	107.5
Initial weight GP1, kg	381.5	420	359	407	412	404	379	335.5
Initial weight IP2, kg	416	447.5	422	489.5	437.5	457.5	413.5	394
Initial weight GP2, kg	.	.	.	.	.	.	.	.
Initial weight IP3, kg	.	.	.	.	.	.	.	.

[a]1 is dairy x beef crossbreed, 2 is dairy breed.

[b]1 is spring-born, moderately high indoor feed intensity with 21 months slaughter age, 2 is autumn-born, low indoor feed intensity and 28 months slaughter age.

[c]1 is not infected, 2 is infected.

[d]EUROP system on a scale 1 = poor, 15 = excellent.

[e]EUROP system on a scale 1 = lean, 15 = fat.

[f]Visually determined in *Musculus longissimus dorsi* between 10th and 11th ribs on a scale 1 = lean and 5 = well marbled.

[g]Right hind quarter.

[h]Commercial cut meat assortment estimated to contain 10% fat.

[i]Commercial cut meat assortment estimated to contain 23% fat.

[j]High-value retail cuts; strip loin, fillet, topside, outside round, eye of round, top rump, and rump steak

[k]Relative to the oldest animal in that production system group.

Table S2 cont.

Animal ID	8338	8359	8383	8398	8336	8352	8371	8382
Date of birth	150606	150618	150629	150706	150604	150614	150625	150629
Pen no.	17	17	17	17	18	18	18	18
Breed[a]	1	1	1	1	2	2	2	2
Production system[b]	1	1	1	1	1	1	1	1
Pair of calves, no	9	10	11	12	9	10	11	12
Parasites[c]	1	1	1	1	1	1	1	1
Weight gain IP1, kg d^{-1}	1.243	1.205	1.070	0.734	1.302	1.115	.	1.005
Weight gain GP1, kg d^{-1}	0.299	0.447	0.641	1.127	0.440	0.662	.	0.680
Weight gain IP2, kg d^{-1}	1.339	1.208	1.339	1.068	1.429	1.071	.	1.182
Weight gain GP2, kg d^{-1}	.	.	.	.	.	.	.	.
Weight gain IP3, kg d^{-1}	.	.	.	.	.	.	.	.
Weight gain total, kg d^{-1}	1.022	1.004	1.040	0.945	1.112	0.980		0.974
Weight at slaughter, kg	684.5	676.0	681.0	607.5	727.0	643.5	.	621.0
Carcass weight, kg	327.8	338.1	323.9	293.5	333.2	299.9	.	289.6
Dressing, %	47.9	50.0	47.6	48.3	45.8	46.6	.	46.6
Conformation, score[d]	6	6	5	6	5	5	.	3
Fatness, score[e]	8	8	7	7	8	7	.	6
Marbling, score[f]	1	2	1	2	3	3	.	2
Weight of HQ[g], kg	86	88	86	77.5	84.5	78.5	.	74
Trim fat, % of HQ	7.67	9.84	6.10	6.87	9.07	7.62	.	7.03
Gr. 2 meat ass.[h], % of HQ	20.7	18.1	20.2	20.0	19.7	19.9	.	19.7
Gr. 3 meat ass.[i], % of HQ	10.3	11.0	11.3	10.8	11.6	12.0	.	10.8
Bone, % of HQ	20.5	19.8	20.1	20.1	20.7	21.2	.	21.9
Retail cuts[j], % of HQ	37.6	38.2	38.9	38.4	35.3	36.4	.	37.4
Slaughter age, d	641	629	618	611	643	633	.	618
Relative age[k], d	52	64	75	82	50	60	.	75
Initial weight IP1, kg	141	142	127.5	105	135.5	122	.	103
Initial weight GP1, kg	417	409.5	365	268	424.5	369.5	.	326
Initial weight IP2, kg	459.5	473	456	428	487	463.5	.	422.5
Initial weight GP2, kg	.	.	.	.	.	.	.	.
Initial weight IP3, kg	.	.	.	.	.	.	.	.

[a]1 is dairy x beef crossbreed, 2 is dairy breed.

[b]1 is spring-born, moderately high indoor feed intensity with 21 months slaughter age, 2 is autumn-born, low indoor feed intensity and 28 months slaughter age.

[c]1 is not infected, 2 is infected.

[d]EUROP system on a scale 1 = poor, 15 = excellent.

[e]EUROP system on a scale 1 = lean, 15 = fat.

[f]Visually determined in *Musculus longissimus dorsi* between 10th and 11th ribs on a scale 1 = lean and 5 = well marbled.

[g]Right hind quarter.

[h]Commercial cut meat assortment estimated to contain 10% fat.

[i]Commercial cut meat assortment estimated to contain 23% fat.

[j]High-value retail cuts; strip loin, fillet, topside, outside round, eye of round, top rump, and rump steak

[k]Relative to the oldest animal in that production system group.

Table S2 cont.

Animal ID	8400	8404	8406	8416	8394	8399	8409	8410
Date of birth	150708	150709	150710	150714	150705	150706	150711	150711
Pen no.	19	19	19	19	20	20	20	20
Breed[a]	1	1	1	1	2	2	2	2
Production system[b]	1	1	1	1	1	1	1	1
Pair of calves, no	13	14	15	16	13	14	15	16
Parasites[c]	2	2	2	2	2	2	2	2
Weight gain IP1, kg d^{-1}	1.146	1.065	1.063	1.029	.	0.885	1.092	1.149
Weight gain GP1, kg d^{-1}	0.356	0.229	0.486	0.239	.	0.405	0.451	0.423
Weight gain IP2, kg d^{-1}	1.189	0.980	1.240	1.202	.	0.929	1.337	1.151
Weight gain GP2, kg d^{-1}	.	.	.	.	.	.	.	.
Weight gain IP3, kg d^{-1}	.	.	.	.	.	.	.	.
Weight gain total, kg d^{-1}	0.961	0.823	0.979	0.889	.	0.779	1.015	0.965
Weight at slaughter, kg	657.5	563.0	659.5	618.5	.	526.0	662.0	625.0
Carcass weight, kg	321.0	276.4	326.8	300.4	.	251.9	295.0	290.1
Dressing, %	48.8	49.1	49.6	48.6	.	47.9	44.6	46.4
Conformation, score[d]	5	4	5	4	.	4	4	4
Fatness, score[e]	9	8	9	8	.	9	9	8
Marbling, score[f]	2	1	1	1	.	3	1	2
Weight of HQ[g], kg	84.4	73.6	86	77.6	.	65.2	75.4	74.6
Trim fat, % of HQ	5.56	6.61	8.75	3.82	.	8.58	8.58	6.50
Gr. 2 meat ass.[h], % of HQ	19.8	20.0	17.9	21.0	.	18.2	18.5	18.9
Gr. 3 meat ass.[i], % of HQ	13.6	12.0	12.5	10.0	.	14.0	12.2	11.2
Bone, % of HQ	19.3	21.9	20.2	23.0	.	20.4	23.6	23.7
Retail cuts[j], % of HQ	38.2	37.4	36.7	38.3	.	35.1	36.2	36.6
Slaughter age, d	637	636	635	631	.	639	634	634
Relative age[k], d	84	85	86	90	.	82	87	87
Initial weight IP1, kg	119.5	102	111.5	120.5	74	90	93.5	84.5
Initial weight GP1, kg	374	338.5	347.5	349	246.5	286.5	336	339.5
Initial weight IP2, kg	424.5	371	416.5	383	313.5	344	400	399.5
Initial weight GP2, kg	.	.	.	.	.	.	.	.
Initial weight IP3, kg	.	.	.	.	.	.	.	.

[a]1 is dairy x beef crossbreed, 2 is dairy breed.
[b]1 is spring-born, moderately high indoor feed intensity with 21 months slaughter age, 2 is autumn-born, low indoor feed intensity and 28 months slaughter age.
[c]1 is not infected, 2 is infected.
[d]EUROP system on a scale 1 = poor, 15 = excellent.
[e]EUROP system on a scale 1 = lean, 15 = fat.
[f]Visually determined in *Musculus longissimus dorsi* between 10th and 11th ribs on a scale 1 = lean and 5 = well marbled.
[g]Right hind quarter.
[h]Commercial cut meat assortment estimated to contain 10% fat.
[i]Commercial cut meat assortment estimated to contain 23% fat.
[j]High-value retail cuts; strip loin, fillet, topside, outside round, eye of round, top rump, and rump steak
[k]Relative to the oldest animal in that production system group.

Table S2 cont.

Animal ID	8419	8423	8488	8491	8413	8414	8425	8476
Date of birth	150715	150717	150814	150817	150712	150712	150717	150807
Pen no.	21	21	21	21	22	22	22	22
Breed[a]	1	1	1	1	2	2	2	2
Production system[b]	2	2	2	2	2	2	2	2
Pair of calves, no	17	18	19	20	17	18	19	20
Parasites[c]	2	2	2	2	2	2	2	2
Weight gain IP1, kg d^{-1}	0.822	1.092	1.306	0.770	0.839	0.933	1.069	0.875
Weight gain GP1, kg d^{-1}	0.299	0.194	0.468	0.651	0.137	0.532	0.482	0.183
Weight gain IP2, kg d^{-1}	0.765	0.931	0.892	0.521	0.465	0.645	0.650	0.733
Weight gain GP2, kg d^{-1}	0.451	0.260	0.383	0.532	0.120	0.438	0.286	0.695
Weight gain IP3, kg d^{-1}	1.795	2.125	2.179	0.848	.	1.357	1.884	0.732
Weight gain total, kg d^{-1}	0.698	0.769	0.887	0.627	.	0.704	0.736	0.655
Weight at slaughter, kg	635	739	825	581	.	652	683	597.5
Carcass weight, kg	289.1	362.6	388.1	292.0	.	294	315.6	275.4
Dressing, %	45.6	49.1	47.1	50.3	.	45.092	46.2	46.1
Conformation, score[d]	5	7	7	5	.	4	4	4
Fatness, score[e]	4	7	7	5	.	5	7	5
Marbling, score[f]	1	1	1	1	.	2	1	1
Weight of HQ[g], kg	73.8	92.8	96.8	71.4	.	72.4	77.4	68
Trim fat, % of HQ	4.58	8.73	8.64	4.85	.	8.12	9.07	6.71
Gr. 2 meat ass.[h], % of HQ	27.2	23.4	23.8	25.6	.	22.8	24.5	24.1
Gr. 3 meat ass.[i], % of HQ	4.8	7.0	6.9	5.7	.	6.0	5.3	5.7
Bone, % of HQ	21.5	19.9	20.5	21.0	.	22.2	21.3	22.0
Retail cuts[j], % of HQ	40.2	39.2	38.6	41.1	.	39.8	38.0	40.1
Slaughter age, d	861	859	831	828	.	864	859	838
Relative age[k], d	3	5	33	36	.	0	5	26
Initial weight IP1, kg	131	184.5	185	129	112.5	125	131.5	107
Initial weight GP1, kg	256	350.5	383.5	246	263.5	293	324	264.5
Initial weight IP2, kg	298.5	378	450	338.5	283	368.5	392.5	290.5
Initial weight GP2, kg	464.5	580	643.5	451.5	384	508.5	533.5	449.5
Initial weight IP3, kg	534	620	702.5	533.5	402.5	576	577.5	556.5

[a]1 is dairy x beef crossbreed, 2 is dairy breed.

[b]1 is spring-born, moderately high indoor feed intensity with 21 months slaughter age, 2 is autumn-born, low indoor feed intensity and 28 months slaughter age.

[c]1 is not infected, 2 is infected.

[d]EUROP system on a scale 1 = poor, 15 = excellent.

[e]EUROP system on a scale 1 = lean, 15 = fat.

[f]Visually determined in *Musculus longissimus dorsi* between 10th and 11th ribs on a scale 1 = lean and 5 = well marbled.

[g]Right hind quarter.

[h]Commercial cut meat assortment estimated to contain 10% fat.

[i]Commercial cut meat assortment estimated to contain 23% fat.

[j]High-value retail cuts; strip loin, fillet, topside, outside round, eye of round, top rump, and rump steak

[k]Relative to the oldest animal in that production system group.

Table S2 cont.

Animal ID	8499	8510	8525	8533	8485	8492	8511	8515
Date of birth	150820	150828	150904	150913	150812	150817	150828	150830
Pen no.	23	23	23	23	24	24	24	24
Breed[a]	1	1	1	1	2	2	2	2
Production system[b]	2	2	2	2	2	2	2	2
Pair of calves, no	21	22	23	24	21	22	23	24
Parasites[c]	2	2	2	2	2	2	2	2
Weight gain IP1, kg d^{-1}	1.158	0.865	0.888	1.049	0.789	1.217	0.901	1.026
Weight gain GP1, kg d^{-1}	0.634	0.197	0.694	0.644	0.715	0.507	0.461	0.585
Weight gain IP2, kg d^{-1}	0.813	0.548	0.680	0.583	0.696	0.712	0.645	0.631
Weight gain GP2, kg d^{-1}	0.370	0.831	0.692	0.802	0.403	0.588	0.295	0.601
Weight gain IP3, kg d^{-1}	1.685	1.446	1.482	1.738	1.351	1.696	1.321	1.565
Weight gain total, kg d^{-1}	0.856	0.705	0.817	0.864	0.732	0.860	0.666	0.801
Weight at slaughter, kg	764.5	642	705.5	768	633	772	595	681
Carcass weight, kg	363.6	308.7	331.7	362.6	282.2	352.3	272	295
Dressing, %	47.6	48.1	47.0	47.2	44.6	45.6	45.7	43.3
Conformation, score[d]	7	7	6	7	5	4	4	4
Fatness, score[e]	7	7	7	8	7	8	7	6
Marbling, score[f]	1	1	1	2	2	3	2	1
Weight of HQ[g], kg	93.6	79.8	84.8	94.4	72.4	88.4	69.2	75.2
Trim fat, % of HQ	8.31	6.37	7.74	8.22	8.20	10.02	10.55	7.69
Gr. 2 meat ass.[h], % of HQ	23.3	25.7	24.6	26.8	23.3	24.0	24.1	21.7
Gr. 3 meat ass.[i], % of HQ	6.4	5.6	5.0	5.0	5.6	5.2	5.7	7.0
Bone, % of HQ	19.7	20.1	21.0	18.7	21.3	21.4	19.2	21.8
Retail cuts[j], % of HQ	39.0	40.0	38.2	40.2	37.9	36.1	36.3	37.4
Slaughter age, d	853	845	838	829	861	856	845	843
Relative age[k], d	39	47	54	63	31	36	47	49
Initial weight IP1, kg	123.5	114	93.5	121	85	127.5	96	81
Initial weight GP1, kg	299.5	245.5	228.5	280.5	205	312.5	233	237
Initial weight IP2, kg	389.5	273.5	327	372	306.5	384.5	298.5	320
Initial weight GP2, kg	566	392.5	474.5	498.5	457.5	539	438.5	457
Initial weight IP3, kg	623	520.5	581	622	519.5	629.5	484	549.5

[a]1 is dairy x beef crossbreed, 2 is dairy breed.

[b]1 is spring-born, moderately high indoor feed intensity with 21 months slaughter age, 2 is autumn-born, low indoor feed intensity and 28 months slaughter age.

[c]1 is not infected, 2 is infected.

[d]EUROP system on a scale 1 = poor, 15 = excellent.

[e]EUROP system on a scale 1 = lean, 15 = fat.

[f]Visually determined in *Musculus longissimus dorsi* between 10th and 11th ribs on a scale 1 = lean and 5 = well marbled.

[g]Right hind quarter.

[h]Commercial cut meat assortment estimated to contain 10% fat.

[i]Commercial cut meat assortment estimated to contain 23% fat.

[j]High-value retail cuts; strip loin, fillet, topside, outside round, eye of round, top rump, and rump steak

[k]Relative to the oldest animal in that production system group.

Table S2 cont.

Animal ID	8540	8560	8577	8587	8522	8545	8559	8562
Date of birth	150913	150921	151003	151008	150902	150915	150920	150922
Pen no.	25	25	25	25	26	26	26	26
Breed[a]	1	1	1	1	2	2	2	2
Production system[b]	2	2	2	2	2	2	2	2
Pair of calves, no	25	26	27	28	25	26	27	28
Parasites[c]	1	1	1	1	1	1	1	1
Weight gain IP1, kg d^{-1}	1.028	0.848	1.219	1.360	0.833	0.974	1.104	0.953
Weight gain GP1, kg d^{-1}	0.835	0.937	0.746	0.782	0.683	0.859	0.648	0.637
Weight gain IP2, kg d^{-1}	0.700	0.661	0.689	0.737	0.532	0.606	0.668	0.696
Weight gain GP2, kg d^{-1}	1.136	1.175	1.169	1.448	0.721	0.692	0.347	0.370
Weight gain IP3, kg d^{-1}	1.009	1.161	1.259	1.362	1.063	1.116	1.531	1.482
Weight gain total, kg d^{-1}	0.804	0.815	0.798	0.857	0.725	0.802	0.788	0.771
Weight at slaughter, kg	749	693	746	816.5	685.5	710.5	715.5	683.5
Carcass weight, kg	369	339.6	367	417.5	308.7	333.7	321.4	304.3
Dressing, %	49.3	49.0	49.2	51.1	45.0	47.0	44.9	44.5
Conformation, score[d]	6	7	7	6	4	4	4	4
Fatness, score[e]	8	8	7	8	7	8	8	7
Marbling, score[f]	3	2	2	2	1	1	2	2
Weight of HQ[g], kg	91	86.8	94.6	105.6	77.4	86.6	81.6	76.8
Trim fat, % of HQ	8.88	8.53	8.12	9.51	7.52	8.73	8.92	8.18
Gr. 2 meat ass.[h], % of HQ	23.5	25.5	24.4	23.7	23.0	21.2	24.6	26.0
Gr. 3 meat ass.[i], % of HQ	6.5	7.6	6.9	6.1	7.1	7.6	6.3	7.4
Bone, % of HQ	20.1	18.1	18.5	19.5	22.3	20.8	20.3	19.5
Retail cuts[j], % of HQ	32.6	36.8	38.9	38.1	36.1	38.3	36.7	36.1
Slaughter age, d	857	849	837	832	868	855	850	848
Relative age[k], d	63	71	83	88	52	65	70	72
Initial weight IP1, kg	175	111	176.5	204.5	163	132	147.5	127.5
Initial weight GP1, kg	266.5	186.5	285	325.5	243	225.5	253.5	219
Initial weight IP2, kg	385	319.5	391	436.5	340	347.5	345.5	309.5
Initial weight GP2, kg	537	463	540.5	596.5	455.5	479	490.5	460.5
Initial weight IP3, kg	636	563	605	664	566.5	585.5	544	517.5

[a]1 is dairy x beef crossbreed, 2 is dairy breed.

[b]1 is spring-born, moderately high indoor feed intensity with 21 months slaughter age, 2 is autumn-born, low indoor feed intensity and 28 months slaughter age.

[c]1 is not infected, 2 is infected.

[d]EUROP system on a scale 1 = poor, 15 = excellent.

[e]EUROP system on a scale 1 = lean, 15 = fat.

[f]Visually determined in *Musculus longissimus dorsi* between 10th and 11th ribs on a scale 1 = lean and 5 = well marbled.

[g]Right hind quarter.

[h]Commercial cut meat assortment estimated to contain 10% fat.

[i]Commercial cut meat assortment estimated to contain 23% fat.

[j]High-value retail cuts; strip loin, fillet, topside, outside round, eye of round, top rump, and rump steak

[k]Relative to the oldest animal in that production system group.

Table S2 cont.

Animal ID	8606	8620	8642	8646	8592	8600	8626	8636
Date of birth	151016	151026	151104	151107	151011	151014	151027	151101
Pen no.	27	27	27	27	28	28	28	28
Breed[a]	1	1	1	1	2	2	2	2
Production system[b]	2	2	2	2	2	2	2	2
Pair of calves, no	29	30	31	32	29	30	31	32
Parasites[c]	1	1	1	1	1	1	1	1
Weight gain IP1, kg d^{-1}	1.135	1.185	1.006	0.949	1.177	1.182	0.906	1.238
Weight gain GP1, kg d^{-1}	0.803	0.817	0.884	0.803	0.588	0.655	0.338	0.796
Weight gain IP2, kg d^{-1}	0.643	0.825	0.654	0.657	0.654	0.601	0.523	0.730
Weight gain GP2, kg d^{-1}	0.581	0.594	0.578	0.779	0.581	0.695	0.545	0.526
Weight gain IP3, kg d^{-1}	0.449	0.891	0.833	0.963	0.776	0.602	0.874	1.133
Weight gain total, kg d^{-1}	0.681	0.832	0.759	0.804	0.717	0.704	0.610	0.991
Weight at slaughter, kg	667.5	779.5	703	723.5	672	661.5	564	749
Carcass weight, kg	354.8	407.7	364.6	365.1	323.4	318	244	338.6
Dressing, %	53.2	52.3	51.9	50.5	48.1	48.1	43.3	45.2
Conformation, score[d]	6	7	6	6	4	3	2	3
Fatness, score[e]	7	8	8	6	7	7	6	6
Marbling, score[f]	3	3	2	1	5	3	2	1
Weight of HQ[g], kg	89.4	102.6	93.2	92.8	80	78.4	62.2	83.4
Trim fat, % of HQ	6.80	7.58	6.14	6.94	5.98	6.68	6.62	6.67
Gr. 2 meat ass.[h], % of HQ	19.3	18.5	19.7	23.0	18.1	17.9	19.3	21.1
Gr. 3 meat ass.[i], % of HQ	11.1	11.8	11.4	7.6	12.4	14.0	10.4	10.4
Bone, % of HQ	21.3	19.9	21.2	20.2	22.4	21.4	23.8	22.3
Retail cuts[j], % of HQ	38.2	38.3	37.9	38.7	36.9	36.2	35.7	35.6
Slaughter age, d	859	849	840	837	864	861	848	843
Relative age[k], d	96	106	115	118	91	94	107	112
Initial weight IP1, kg	157.5	156.5	134.5	121	130	129	103	128.5
Initial weight GP1, kg	258.5	262	224	205.5	243	242.5	190	230
Initial weight IP2, kg	372.5	378	349.5	319.5	326.5	335.5	238	343
Initial weight GP2, kg	512	557	491.5	462	468.5	466	351.5	501.5
Initial weight IP3, kg	601.5	648.5	580.5	582	558	573	435.5	582.5

[a]1 is dairy x beef crossbreed, 2 is dairy breed.

[b]1 is spring-born, moderately high indoor feed intensity with 21 months slaughter age, 2 is autumn-born, low indoor feed intensity and 28 months slaughter age.

[c]1 is not infected, 2 is infected.

[d]EUROP system on a scale 1 = poor, 15 = excellent.

[e]EUROP system on a scale 1 = lean, 15 = fat.

[f]Visually determined in *Musculus longissimus dorsi* between 10th and 11th ribs on a scale 1 = lean and 5 = well marbled.

[g]Right hind quarter.

[h]Commercial cut meat assortment estimated to contain 10% fat.

[i]Commercial cut meat assortment estimated to contain 23% fat.

[j]High-value retail cuts; strip loin, fillet, topside, outside round, eye of round, top rump, and rump steak

[k]Relative to the oldest animal in that production system group.
